# Predicting the Subjective Health Experience of Older Adults: A Modelling Approach

**DOI:** 10.1155/jare/9997013

**Published:** 2025-11-29

**Authors:** Damien S. E. Broekharst, Sjaak Bloem, Eline J. Mertens, Nathascha Hanzen, Michel van Agthoven

**Affiliations:** ^1^Center for Marketing & Supply Chain Management, Nyenrode Business University, Breukelen, the Netherlands; ^2^Janssen-Cilag B.V., Johnson & Johnson, Breda, the Netherlands

**Keywords:** acceptance, control, frailty, older adults, subjective health experience, vitality

## Abstract

**Introduction:**

As people age, they increasingly face physical and cognitive decline, which can negatively affect their subjective health experience. To improve subjective health experience among older adults, it is crucial to understand its key predictors. Current literature points to frailty and vitality from geriatric research as well as acceptance and control from psychological science as key predictors. However, until now, they have never been incorporated in one composite model. Therefore, this study aims to develop, validate and differentiate a composite model integrating these predictors.

**Methods:**

An online questionnaire covering sample characteristics and instruments on frailty, vitality, acceptance, control and subjective health experience was distributed among a sample of 753 older adults recruited from a research panel. Data were analysed using descriptive, reliability, validity and model statistics.

**Results:**

Frailty shows modest to moderate negative relationships with acceptance, control and subjective health experience and a stronger negative relationship with vitality. Vitality relates moderately positively to control and subjective health experience and modestly to acceptance. Control has a strong positive relationship with acceptance and a modest one with subjective health experience. Acceptance shows a moderate positive relationship with subjective health experience. Frailty has a moderate significant negative indirect relationship with subjective health experience, through several modest but significant pathways involving vitality, acceptance and control combined or alone. Pathways with control alone are modest and nonsignificant. The models explain 41.5%–52.6% of the variance in subjective health experience.

**Conclusion:**

It might be worthwhile to consider deploying the key concepts of the model, such as frailty, vitality, acceptance or control, as starting points for the development of future interventions concerning the subjective health experience of older adults. Such interventions may target frailty and promote vitality through tailored support, while aligning delivery with older adults' needs for control and acceptance.

## 1. Introduction

As individuals progress into later stages of life, they are ever more frequently beset by an array of physiological and cognitive impairments [1–3]. Such decline may exert an adverse influence upon the subjective health experience of these older adults [3, 4]. Subjective health experience is defined as an individual's experience of physical and mental functioning while living their life the way they want to, within the actual constraints and limitations of individual existence [5, 6]. In order to aid older adults in preserving favourable subjective health experience in later life, it is imperative to attain a nuanced understanding of the factors that predict this experience [7, 8]. Within current scholarly discourse, several hypothetical propositions regarding the predictors of subjective health experience in older adults have been advanced [9–13]. These proposed predictors of subjective health experience in older adults largely emanate from two distinct domains of scientific inquiry, namely, geriatric research and psychological science [9–13].

Within the field of geriatric research, two main predictors of subjective health experience in older adults have been proposed, namely, frailty and vitality [9–11]. Both concepts are commonly employed in order to map the age-related health state of an older adult making them useful for diagnostic purposes and prognostic research [14–17]. Frailty conveys a clinically identifiable state of increased vulnerability stemming from age-related deterioration in reserve, function and homoeostatic capacity among several physiological systems such that the ability to manage routine or acute stressors significantly diminishes [14, 15]. Vitality expresses an individual's inherent capacity and inner drive to independently sustain a mode of existence that supports living, growing and developing in a vigorous, dynamic and spirited fashion [16, 17]. It is also worth noting that frailty may likewise function as a valuable proxy for gauging vitality in older adults as it might be regarded as a fundamental constituent of overall vitality [18].

Within the field of psychological science, also two main predictors of subjective health experience in older adults have been proposed, namely, acceptance and control [13]. Both concepts characterise the way older adults perceive and respond to their particular age-related health state making them useful for differentiating treatment and tailoring interventions [13]. Acceptance conveys the extent to which individuals are capable of perceiving and embracing their health condition as an intrinsic element of their personal identity and lived experience [5, 6, 13]. Control expresses the extent to which individuals believe themselves to be capable of exerting meaningful influence over the course, management and outcomes of their own health condition [5, 6, 13]. It should also be recognised that control and acceptance are closely interrelated concepts which have been repeatedly and effectively employed as mediating mechanisms between specific independent variables, such as health perception, health consciousness or health-related quality of life and subjective health experience [19].

**Figure 1:**
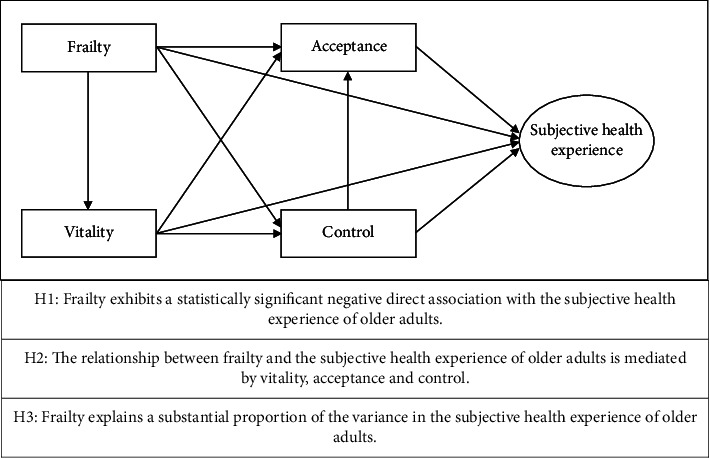
Composite model and underlying hypotheses.

While some of the aforementioned predictors have been empirically examined in relation to subjective health experience, these investigations have been limited, sporadic and fragmented [9–13]. Consequently, few coordinated efforts have been undertaken to integrate these geriatric and psychological predictors together into a composite model for understanding subjective health experience in older adults. Accordingly, the present study seeks to conceptualise, operationalise and empirically validate a composite model integrating both geriatric and psychological predictors in order to predict subjective health experiences in older adults guided by three underlying hypotheses (see Figure 1). This study further also differentiates this composite model across key demographic variables yielding deeper insights into subgroup variations among older adults. Through the examination of this composite model, it will become evident whether it is advantageous for healthcare professionals and other stakeholders to incorporate its key concepts, such as frailty, vitality, acceptance or control, as foundational elements in the design and implementation of future interventions.

## 2. Methods

### 2.1. Design, Procedure and Participants

In order to explore this composite model for predicting subjective health experience in older adults, a quantitative cross-sectional research design was implemented using online questionnaires. These online questionnaires were distributed among a sample of 753 older adults (> 67 years of age) recruited from a preexisting research panel of older adults who were engaged in previous research of the Dutch National Foundation for Older Adults. Only participants who provided explicit informed consent and affirmed no objection to the use of their responses for research purposes were included in the study. These participants were additionally subjected to a verification process to identify and exclude any instances of duplicate panel membership.

### 2.2. Questionnaire, Items and Scales

Sample characteristics such as age, gender, education level, living arrangement and residential area were delineated using individual items. These items were assessed on nominal scales employing dichotomous response categories or ordinal scales with ascending response categories. Frailty was determined using the Groningen Frailty Index (GFI) instrument [20]. This GFI instrument consists of 15 items covering three dimensions, namely, ‘daily activities,' ‘psychosocial functioning' and ‘health problems' [20]. A dichotomous response scale was employed to assess these items and dimensions yielding a cumulative score ranging from 0, indicating an absence of frailty, to 15, reflecting a high degree of frailty [20, 21]. Vitality was assessed using the Vita-16 questionnaire [16]. This Vita-16 questionnaire encompasses 16 items covering three subscales, namely, ‘energy,' ‘motivation' and ‘resilience' [16]. A 7-point Likert scale ranging from 1 = seldom to 7 = always was employed to measure these items and dimensions [16]. The overall vitality score was calculated as weighted composite assigning proportional weights of 0.4 to the energy subscale, 0.3 to the motivation subscale and 0.3 to the resilience subscale, which is in accordance with previous research [13, 16]. Acceptance and control were assessed using their corresponding scales within the Bloem & Stalpers questionnaire [5, 6]. Both concepts were measured using three items accompanied by a 7-point Likert scale ranging from 1 = fully disagree to 7 = fully agree [5, 6]. Subjective health experience was assessed using the Bloem & Stalpers ladder scale [5, 6]. Scores were determined on an 11-level ladder scale ranging from Level 0 = worst day of previous month to Level 11 = best day of previous month [5, 6].

### 2.3. Analysis, Interpretation and Software

Sample characteristics were described using descriptive statistics. These nominal and ordinal variables were expressed in percentages. Questionnaire characteristics were described using composite reliability and construct validity statistics. The composite reliability of the instruments was assessed using Cronbach's alpha (*α*) and rho_*a* and rho_*c* coefficients, which indicated reliability if their value exceeds the 0.70 threshold [22, 23]. The construct validity of the instruments was evaluated through factor analysis in which the underlying factorial structure of the scales was both confirmed and refined if necessary by excluding items exhibiting factor loadings (FL) below 0.50 and removing those demonstrating cross-loadings on multiple factors [22, 23]. Model characteristics were assessed through partial least squares structural equation modelling (PLS-SEM) with results reported using effect sizes, significance levels and explained variance. The effect sizes of relationships between model variables are interpreted using standardised beta (*β*) coefficients, which can be considered modest below 0.25, moderate between 0.25 and 0.50 and large above 0.50 [22, 23]. The significance levels of the aforementioned relationships are analysed using *p* values, which are considered significant if they are lower than 0.05 [22, 23]. The explained variance of independent variables in the dependent variable was described using *R*-squared (*R*^2^) coefficients, which can be considered modest below 0.25, moderate between 0.25 and 0.50 and large above 0.50 [22, 23]. Sample characteristics were extracted using software package IBM SPSS Statistics Version 27 [24]. Questionnaire and model characteristics were analysed using software package SmartPLS Version 4.0 [22]. Grammar and readability of this article was enhanced using ChatGPT.

## 3. Results

### 3.1. Sample Characteristics

The sample analysed in this study closely resembled the older Dutch population in terms of age, gender, education level, household size and residential area, while also representing a group characterised by relatively independent living arrangements [25–31]. The selection of sample characteristics was guided by prevailing policy standards and previous research [32]. The particular characteristics of the sample are displayed in Table 1.

The models analysed in this study will be stratified according to the demographic variables outlined in Table 1 with the exception of living arrangement. This particular variable exhibits such pronounced asymmetry in its distribution that the subgroup representing independent living mirrors the overall model to a considerable degree, while the cohort encompassing alternative living arrangements is of insufficient size to yield results of requisite reliability and validity.

### 3.2. Instrument Characteristics

The composite reliability of the instruments on frailty (*α* = 0.724; rho_*a* = 0.780; rho_*c* = 0.769), vitality (*α* = 0.958; rho_*a* = 0.963; rho_*c* = 0.962), acceptance (*α* = 0.923; rho_*a* = 0.924; rho_*c* = 0.951) and control (*α* = 0.882; rho_*a* = 0.895; rho_*c* = 0.927) can be deemed sufficient as these coefficients exceeded the 0.70 threshold. The instruments assessing vitality (FL = 0.623–0.840), acceptance (FL = 0.924–0.937) and control (FL = 0.860–0.925) demonstrated satisfactory construct validity with all FL surpassing the 0.50 threshold and each item loading exclusively onto its intended latent construct without indications of cross-loading. For the instrument on frailty, no construct validity could be determined as it represents an index measure composed of discrete and conceptually uncorrelated indicators lacking an underlying factorial structure by design. For the instrument on subjective health experience, neither composite reliability nor construct validity could be determined as it is comprised of a single value and is not multidimensional in nature.

### 3.3. Model Characteristics

The composite model for predicting subjective health experiences in older adults presented in this study will be elucidated by explicating the direct relationships, indirect relationships and explained variance across the overall model and each of its stratified variants. It should be mentioned that the theoretically assumed relationships in each of these models were relatively strongly supported by empirical evidence, while this was not the case when these relationships were inverted decreasing the probability of reverse causality.

#### 3.3.1. Direct Relationships

From the analysis (see Table 2) emerges that frailty shows a modest significant negative relationship with acceptance in the overall (*β* = −0.166) and differentiated models (*β* = −0.146 to −0.181). Frailty has a modest significant negative relationship with control in the overall (*β* = −0.155) and differentiated models (*β* = −0.096 to −0.205) except among those less educated in which it was not significant. Frailty demonstrates a modest to moderate significant negative relationship with subjective health experience in the overall (*β* = −0.231) and differentiated models (*β* = −0.196 to −0.280). Frailty indicates a large significant negative relationship with vitality in the overall (*β* = −0.632) and differentiated models (*β* = −0.582–−0.664). Vitality shows a moderate significant positive relationship with control in the overall (*β* = 0.439) and differentiated models (*β* = 0.383–0.481). Vitality exhibits a modest significant positive relationship with acceptance in the overall (*β* = 0.099) and differentiated models for those under 75, less educated, cohabiting and female, but not in others (*β* = 0.082–0.128). Vitality exhibited a moderate significant positive relationship with subjective health experience in the overall (*β* = 0.351) and differentiated models (*β* = 0.297–0.402). Control has a large significant positive relationship with acceptance in the overall (*β* = 0.646) and differentiated models (*β* = 0.625–0.666). Control demonstrates a modest nonsignificant negative relationship with subjective health experience in the overall (*β* = −0.074) and differentiated models (*β* = −0.149–0.016) except among those above 75 and female in which it was significant and among males in which it was positive. Acceptance has a modest to moderate significant positive relationship with subjective health experience in the overall (*β* = 0.292) and differentiated models (*β* = 0.243–0.349).

#### 3.3.2. Indirect Relationships

From the analysis (see Table 2) stems that frailty has a moderate significant negative indirect relationship with subjective health experience in the overall (*β* = −0.338) and differentiated models (*β* = −0.312 to −0.351). This overall indirect relationship reflects the cumulative association of all intermediary pathways linking frailty to subjective health experience. One such pathway involves vitality through which frailty shows a modest significant negative indirect relationship with subjective health experience in the overall (*β* = −0.222) and differentiated models (*β* = −0.197 to −0.243). Another pathway involves vitality and acceptance through which frailty shows a modest significant negative indirect relationship with subjective health experience in the overall (*β* = −0.018) and differentiated models for those under 75 and female, but not in others (*β* = −0.013 to −0.022). A third pathway involves vitality, control and acceptance through which frailty shows a modest significant negative indirect relationship with subjective health experience in the overall (*β* = −0.052) and differentiated models (*β* = −0.036 to −0.069). A fourth pathway involves acceptance through which frailty shows significant negative indirect relationship with subjective health experience in the overall (*β* = −0.049) and differentiated models (*β* = −0.036 to −0.063) except among males in which it was not significant. A fifth pathway involves control and acceptance through which frailty shows a modest significant negative indirect relationship with subjective health experience in the overall (*β* = −0.029) and differentiated models (*β* = −0.016 to −0.047) except among those under 75, less educated and male in which it was not significant. A sixth pathway involves control through which frailty shows a modest nonsignificant positive indirect relationship with subjective health experience in the overall (*β* = 0.012) and differentiated models (*β* = −0.003–0.030) except among males in which it was negative. A seventh pathway involves vitality and control through which frailty shows a modest nonsignificant positive indirect relationship with subjective health experience in the overall (*β* = 0.021) and differentiated models (*β* = −0.004–0.041) except among those above 75 and female in which it was significant or among males in which it was negative.

#### 3.3.3. Explained Variance

From the analysis (see Table 2) stems that all models explain between 41.5% and 52.6% of variance in subjective health experience. The overall model accounts for 47.5% of variance in subjective health experience. When stratified by age, this model accounts for 52.6% of the variance in subjective health experience among older adults below 75 and a comparatively lower 41.5% among older adults above 75. When stratified by gender, this model accounts for 46.9% of the variance in subjective health experience among male older adults and 47.4% among female older adults. When stratified by education level, this model accounts for 47.4% of the variance in subjective health experience among older adults with lower education and 45.9% among older adults with higher education. When stratified by household size, this model accounts for 43.7% of the variance in subjective health experience among older adults living alone and a comparatively higher 51.5% among older adults living together. When segmented by residential area, this model accounts for 49.8% of the variance in subjective health experience among older adults living in villages and 46.9% among older adults living in cities.

## 4. Discussion

The present study conceptualised, operationalised, differentiated and empirically validated a composite model integrating geriatric and psychological predictors in order to predict subjective health experiences in older adults. The findings of this study show that frailty has modest to moderate negative relationships with acceptance, control and subjective health experience, while its negative relationship with vitality is larger. The results also indicate that vitality shows moderate positive relationships with control and subjective health experience, while its positive relationship with acceptance is modest. The findings subsequently show that control has a strong positive relationship with acceptance and a modest one with subjective health experience. The analysis further suggests that acceptance generally shows a moderate positive relationship with subjective health experience. The findings additionally suggest that frailty has a moderate significant negative indirect relationship with subjective health experience reflecting the accumulation of several intermediary pathways. Key intermediary pathways include vitality, vitality with acceptance, vitality with control and acceptance, acceptance alone and control with acceptance, which are all modest and significant. Pathways involving control alone show modest nonsignificant positive relationships with subgroup variations. The composite model explains between 41.5% and 52.6% of the variance in subjective health experience across its differentiated variants. From these findings, three principal insights may be discerned.

The first principal insight concerns the added value of geriatric predictors in the composite model. The negative relationship between frailty and subjective health experience appears valid as deteriorating physical, mental and social functioning likely reduces the subjective experience of health [33, 34]. The negative relationships of frailty with control and acceptance seem plausible as physical, mental and social impairments may necessitate greater support leading to a loss of autonomy and difficulty adapting to a changed reality [35–37]. The negative relationship between frailty and vitality appears logical as physical, mental and social decline may drain energy, motivation and resilience necessary for a fulfilling life [17]. The positive relationship between vitality and subjective health experience seems well founded as greater energy, motivation and resilience likely enhances the subjective experience of health [38]. The positive relationships of vitality with control and to a lesser extent acceptance seem defensible as increased energy, motivation and resilience may support personal agency and adaptation to an altered reality [39, 40]. Vitality also serves a mediating role between frailty and subjective health as physical, mental and social difficulties may deplete energy, motivation and resilience harming the subjective experience of health [17]. These robust and coherent relationships suggest that it may be prudent to include geriatric predictors in the composite model.

The second principal insight concerns the added value of psychological predictors in the composite model. The positive relationship between acceptance and subjective health experience seems well supported as an enhanced capacity for reconciliation with a particular health condition may enhance the subjective experience of health [41–43]. The positive relationship between control and acceptance also appears justifiable as the exercise of meaningful agency over a health condition may facilitate its integration into personal identity and lived experience [41–43]. The modest and often nonsignificant negative relationship between control and subjective health experience constitutes a notable anomaly in light of the reasonable expectation that exercising meaningful agency over a particular health condition would ostensibly enhance the subjective experience of health [41–43]. Nevertheless, this intriguing anomaly does not detract from the valuable role of control in the composite model as it also demonstrates a meaningful mediating influence between frailty and subjective health experience in conjunction with acceptance, which appears plausible as the progressive deterioration in physical, mental and social functioning may compromise the capacity for personal agency impeding the acceptance required to establish and preserve a favourable subjective experience of health [41–43]. These nuanced and conceptually rich relationships suggest that it may also be prudent to include psychological predictors in the composite model.

The third principal insight concerns the noteworthy anomaly regarding the concept of control in the composite model. While the dominant view in current scientific discourse holds that maintaining an internal locus of control regarding one's health enhances subjective health experience, alternative studies propose a different perspective that may plausibly explain this anomaly regarding control [44–46]. These studies contend that a significant drawback of an internal locus of control is the inherent responsibility to maintain it, which becomes increasingly arduous for older adults as their age progresses and their level of physical, mental and social fortitude declines [44–46]. The persistent endeavour to uphold an internal locus of control may impose significant psychological strain on older adults leading to heightened stress and anxiety, which may contribute to a diminished subjective health experience [44–46]. This interpretation finds further corroboration in the findings of this study, which reveal a progressively negative and statistically significant relationship between control and subjective health experience when contrasting individuals below and above 75. Comparable anomalies may likewise arise in other contexts characterised by diminished personal agency, such as among individuals afflicted by debilitating conditions or chronic illnesses [19]. These findings call for a more nuanced understanding of control in later life recognising both its potential benefits and its possible psychological burdens.

### 4.1. Strengths and Limitations

This study demonstrates several strengths alongside some potential limitations. A key strength of this study resides in the substantial sample size attained, which enhances the generalisability and representativeness of the findings. Another key strength of this study lies in the high reliability and validity of the measurement instruments employed, which strengthens the veracity and precision of the results. A potential limitation of this study lies in its exclusively Dutch sample and context compounded by a bias toward respondents who live independently and have digital access, which may lead to the distortion or misrepresentation of the findings. Another potential limitation of this study stems from its cross-sectional design and absence of longitudinal data, which inherently restricts the ability to capture causal inferences, temporal dynamics and potential shifts in the relationships between variables over time.

### 4.2. Practical Implications

The findings of this study yield several salient implications for practical application. Grounded in the composite model and its associated predictors, comprehensive interventions may be devised to ameliorate the subjective health experience of older adults. Such interventions could be oriented towards mitigating frailty and fostering vitality (e.g., counselling, coaching and training) based on the specific impairments of an older adult, while the delivery of these interventions (e.g., patient–professional communication, shared decision-making and information provision) is attuned to their particular psychological needs for control and acceptance. Ultimately, this tailored approach holds the potential to meaningfully enrich the subjective health experience within this older population.

### 4.3. Future Research

Four principal avenues for future research could be identified. The first avenue for future research concerns the further differentiation of this model along additional salient demographic dimensions, such as annual income and personality traits. The second avenue for future research entails the validation of this model in different populations of older adults in order to comprehend their subjective health experience, such as older adults living in long-term care facilities. The third avenue for future research advocates for cross-national validation of the model to explore the influence of geographical and cultural contexts. The fourth avenue for future research calls for a more in-depth investigation of the anomaly concerning the concept of control observed in this study with the aim of elucidating this complex construct and its association with subjective health experience.

## 5. Conclusion

The present study conceptualised, operationalised, differentiated and empirically validated a model integrating both geriatric and psychological predictors in order to predict subjective health experiences in older adults. The analysis suggests that the integration of both types of predictors in a composite model is not only theoretically expedient due to its harmonisation of two distinct perspectives on subjective health experience along with their particular merits but also statistically robust due to its considerable predictive power and the meaningful relationships between variables. In light of these findings, it may be considered advantageous for healthcare professionals and relevant stakeholders to adopt one or more core constructs of the model, such as frailty, vitality, acceptance or control, as foundational elements in the design and implementation of future interventions aimed at enhancing the subjective health experience of older adults.

## Figures and Tables

**Table 1:** Sample characteristics.

**Variables**		

Age	59.4% < 75 years	40.6% > 75 years
Gender	40.6% male	59.4% female
Education level	51.1% lower	48.9% higher
Living arrangement	98.9% independent	1.1% other
Household size	51.3% 1 person	48.7% ≥ 2 person
Residential area	42.0% village	58.0% city

**Table 2:** Model characteristics.

	Overall (*β*)	Age (*β*)		Gender (*β*)		Education level (*β*)		Household size (*β*)		Residential area (*β*)	
		< 75	> 75	Male	Female	Lower	Higher	1 person	≥ 2 person	Village	City
*Direct relationships*											
Acceptance ⟶ subjective health experience	**0.292** ^**∗**^	0.268^∗^	0.292^∗^	0.244^∗^	0.325^∗^	0.267^∗^	0.342^∗^	0.349^∗^	0.243^∗^	0.296^∗^	0.282^∗^
Control ⟶ acceptance	**0.646** ^**∗**^	0.633^∗^	0.655^∗^	0.661^∗^	0.637^∗^	0.633^∗^	0.665^∗^	0.666^∗^	0.625^∗^	0.666^∗^	0.637^∗^
Control ⟶ subjective health experience	**−0.074**	−0.011	−0.149^∗∗^	0.016	−0.137^∗∗^	−0.070	−0.102	−0.105	−0.051	−0.082	−0.063
Frailty ⟶ acceptance	**−0.166** ^**∗**^	−0.164^∗^	−0.181^∗^	−0.146^∗∗^	−0.176^∗^	−0.153^∗^	−0.169^∗^	−0.181^∗^	−0.156^∗^	−0.171^∗^	−0.163^∗^
Frailty ⟶ control	**−0.155** ^**∗**^	−0.121^∗∗^	−0.203^∗^	−0.186^∗∗^	−0.134^∗∗^	−0.096	−0.205^∗^	−0.137^∗∗^	−0.184^∗∗^	−0.173^∗∗^	−0.140^∗∗^
Frailty ⟶ subjective health experience	**−0.231** ^**∗**^	−0.267^∗^	−0.196^∗∗^	−0.214^∗∗^	−0.240^∗^	−0.244^∗^	−0.210^∗^	−0.198^∗∗^	−0.257^∗^	−0.202^∗^	−0.280^∗^
Frailty ⟶ vitality	**−0.632** ^**∗**^	−0.640^∗^	−0.598^∗^	−0.629^∗^	−0.631^∗^	−0.650^∗^	−0.598^∗^	−0.618^∗^	−0.625^∗^	−0.582^∗^	−0.664^∗^
Vitality ⟶ acceptance	**0.099** ^**∗∗**^	0.128^∗∗^	0.082	0.084	0.108^∗∗^	0.114^∗∗^	0.092	0.082	0.118^∗∗^	0.103	0.093
Vitality ⟶ control	**0.439** ^**∗**^	0.452^∗^	0.423^∗^	0.387^∗^	0.479^∗^	0.481^∗^	0.397^∗^	0.481^∗^	0.383^∗^	0.435^∗^	0.443^∗^
Vitality ⟶ subjective health experience	**0.351** ^**∗**^	0.328^∗^	0.388^∗^	0.345^∗^	0.352^∗^	0.355^∗^	0.338^∗^	0.319^∗^	0.389^∗^	0.402^∗^	0.297^∗^

*Indirect relationships*											
Frailty ⟶ subjective health experience^∗∗∗^	**−0.338** ^**∗**^	−0.341^∗^	−0.319^∗^	−0.342^∗^	−0.332^∗^	−0.332^∗^	−0.334^∗^	−0.333^∗^	−0.341^∗^	−0.351^∗^	−0.312^∗^
Frailty ⟶ vitality ⟶ subjective health experience	**−0.222** ^**∗**^	−0.210^∗^	−0.232^∗^	−0.217^∗^	−0.222^∗^	−0.231^∗^	−0.202^∗^	−0.197^∗^	−0.243^∗^	−0.234^∗^	−0.197^∗^
Frailty ⟶ control ⟶ subjective health experience	**0.012**	0.001	0.030	−0.003	0.018	0.007	0.021	0.014	0.009	0.014	0.009
Frailty ⟶ acceptance ⟶ subjective health experience	**−0.049** ^**∗**^	−0.044^∗∗^	−0.053^∗∗^	−0.036	−0.057^∗^	−0.041^∗∗^	−0.058^∗∗^	−0.063^∗^	−0.038^∗∗^	−0.050^∗∗^	−0.046^∗∗^
Frailty ⟶ vitality ⟶ acceptance ⟶ subjective health experience	**−0.018** ^**∗∗**^	−0.022^∗∗^	−0.014	−0.013	−0.022^∗∗^	−0.020	−0.019	−0.018	−0.018	−0.018	−0.017
Frailty ⟶ vitality ⟶ control ⟶ subjective health experience	**0.021**	0.003	0.038^∗∗^	−0.004	0.041^∗∗^	0.022	0.024	0.031	0.012	0.021	0.019
Frailty ⟶ control ⟶ acceptance ⟶ subjective health experience	**−0.029** ^**∗∗**^	−0.021	−0.039^∗∗^	−0.030	−0.028^∗∗^	−0.016	−0.047^∗∗^	−0.032^∗∗^	−0.028^∗∗^	−0.034^∗∗^	−0.025^∗∗^
Frailty ⟶ vitality ⟶ control ⟶ acceptance ⟶ subjective health experience	**−0.052** ^**∗**^	−0.049^∗^	−0.048^∗^	−0.039^∗∗^	−0.063^∗^	−0.053^∗^	−0.054^∗^	−0.069^∗^	−0.036^∗^	−0.050^∗^	−0.053^∗^

Explained variance	**47.5%**	**52.6%**	**41.5%**	**46.9%**	**47.4%**	**47.4%**	**45.9%**	**43.7%**	**51.5%**	**49.8%**	**46.9%**

*Note:* The bold values represent the overall values, which are most important for good interpretation.

^∗∗∗^This indirect relationship reflects the cumulative association of all intermediary pathways linking frailty to subjective health experience.

^∗∗^ *p* ≤ 0.05.

^∗^ *p* ≤ 0.001.

## Data Availability

The dataset used during this study is not publicly available, but it is available from the corresponding author upon reasonable request.
